# Pigmentary Retinopathy and Chronic Subretinal Fluid Associated with Pseudoxanthoma Elasticum and Angioid Streaks

**DOI:** 10.18502/jovr.v17i1.10183

**Published:** 2022-01-21

**Authors:** Matthew R. Starr, Sophie J. Bakri

**Affiliations:** Mayo Clinic Department of Ophthalmology, Rochester, MN, USA

##  PRESENTATION

**Figure 1:**
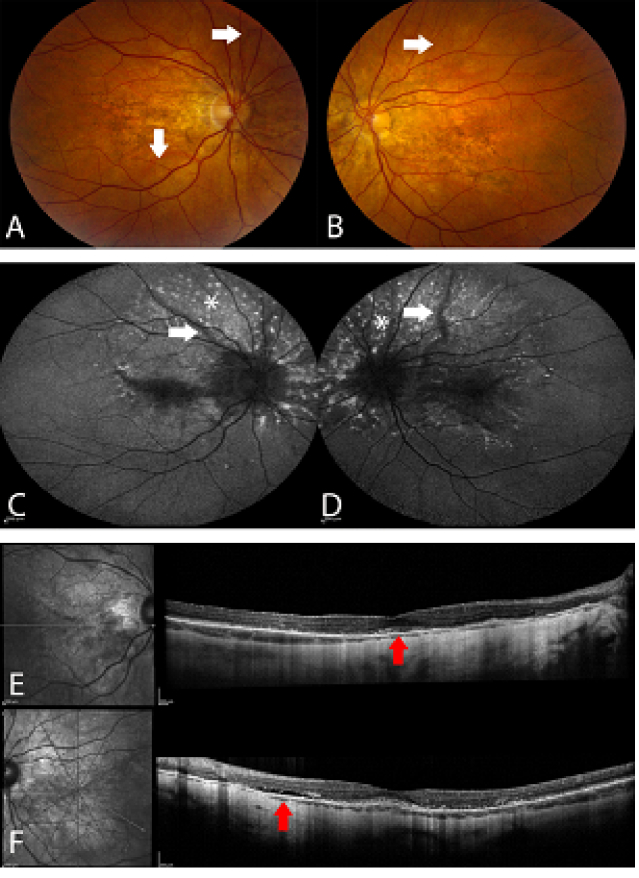
Fundus images (A) with bilateral hyperpigmented lines emanating from the discs, consistent with angioid streaks (white arrows). On FAF (B), the angioid streaks are easily appreciated (white arrows). Also seen are punctate areas of hyper- and hypo-FAF surrounding the disc and within the fovea (asterisks). The hyper-FAF lesions are felt to be accumulations of photoreceptor lipofuscin while the hypo-FAF lesions are thought to be related to subretinal drusenoid deposits. On OCT (C-F), there is evidence of bilateral subretinal fluid (red arrows) consistent with chronic RPE damage that are stable over five years (C & D images taken five years before E & F images).

We present the ophthalmic findings of a 57-year-old female with pseudoxanthoma elasticum who was initially diagnosed in her 20s after a biopsy of abnormal neck lesions. She began to develop visual problems decades later, but currently has no other related medical problems, including any evidence of coronary artery disease. Our patient complained of bilateral metamorphopsia but with preserved visual acuity of 20/25 in both eyes. In addition to the angioid streaks, the patient also had evidence of pigmentary changes at the posterior pole [Figures 1A–1B]. Optical coherence tomography imaging [Figures 1C–1F] revealed the presence of bilateral subretinal fluid, shaggy photoreceptors, and intraretinal hyperreflective material concentrated within the outer retina that was stable for five years [Figures 1 E & 1F].

##  DISCUSSION

Angioid streaks are breaks within a weakened Bruch's membrane. They are deep to the neurosensory retina and typically bilateral, irregular, and emanate from the optic disc. Causes include Ehler–Danlos syndrome, Paget's disease, sickle cell, and other hemoglobinopathies, idiopathic, and pseudoxanthoma elasticum.^[1]^ Vision is typically not affected by angioid streaks unless patients develop choroidal neovascularization with resultant macular edema and retinal hemorrhages.^[2]^ Following our patient for 10 years (5 years with spectral domain OCT), she has never had any evidence of a choroidal neovascular complex on fluorescein angiography and thus it was concluded that the subretinal fluid was the result of malfunctioning retinal pigment epithelium due to the pigmentary changes at the posterior pole, a rare finding first described by Zweifel and colleagues in 2011, who concluded the fluid was due to a similar pathophysiology as pattern dystrophy.^[3]^ Clinicians should be aware of the ophthalmic manifestations of pseudoxanthoma elasticum which include angioid streaks which can lead to choroidal neovascularization and severe vision loss but also pigmentary changes at the posterior pole that can lead to chronic subretinal fluid and a milder effect on visual acuity.

##  Financial Support and Sponsorship

Nil.

##  Conflicts of Interest

There are no conflicts of interest.
